# Exogenous tumor necrosis factor-alpha could induce egress of *Toxoplasma gondii* from human foreskin fibroblast cells

**DOI:** 10.1051/parasite/2017051

**Published:** 2017-11-27

**Authors:** Yong Yao, Miao Liu, Cuiping Ren, Jijia Shen, Yongsheng Ji

**Affiliations:** 1 Department of Microbiology and Parasitology, Anhui Provincial Laboratory of Microbiology and Parasitology; Laboratory of Tropical and Parasitic Diseases Control; Anhui Medical University, Hefei, Anhui 230032 China

**Keywords:** *Toxoplasma gondii*, TNF-α, egress, apoptosis

## Abstract

*Toxoplasma gondii* is an intra-cellular protozoan parasite that can infect almost all nucleated cells, eliciting host immune responses against infection. Host tissue damage is mainly caused by cellular lysis when *T. gondii* egresses from infected cells. However, the effects of cytokines released by host immune cells on egression of *T. gondii* remain elusive. This study aimed to investigate the role of tumor necrosis factor-alpha (TNF-α) on the egress of *T. gondii* from infected human foreskin fibroblast (HFF) cells and to elucidate the underlying mechanisms that regulate TNF-α-induced egress. Using flow cytometry to count tachyzoites of *T. gondii* released into cell culture medium, we found that egress of *T. gondii* from infected HFF cells could be induced by 10 ng/mL TNF-α in a time-dependent manner. Pre-treatment of infected HFF cells with BAPTA-AM to chelate intra-parasitic calcium could greatly inhibit TNF-α-induced egress. Similar results were obtained when using cytochalasin D to block parasite motility before the TNF-α-induced egress assay. In addition, blocking host apoptosis by Z-VAD-FMK could decrease TNF-α induced egress, while blocking necroptosis by necrostatin-1 has little impact on TNF-α-induced egress. The egressed tachyzoites displayed a normal growth rate and lost no virulence. Our results suggest that host cytokines could influence the cellular lytic processes of *T. gondii*, providing new insights into the relationship between host TNF-α and *T. gondii* pathogenesis.

## Abstract (fr)

*Toxoplasma gondii* est un parasite protozoaire intracellulaire qui peut infecter presque toutes les cellules nucléées, provoquant des réponses immunitaires de l'hôte contre l'infection. Les lésions tissulaires de l'hôte sont principalement causées par la lyse cellulaire lorsque *T. gondii* sort des cellules infectées. Cependant, les effets des cytokines libérées par les cellules immunitaires de l'hôte sur la sortie de *T. gondii* étaient peu connus. Cette étude vise à étudier le rôle du facteur de nécrose tumorale alpha (TNF-α) sur la sortie de *T. gondii* de cellules infectées de fibroblastes de prépuce humain (FPH) et à élucider les mécanismes sous-jacents qui régulent la sortie induite par le TNF-α. En utilisant la cytométrie de flux pour compter les tachyzoïtes de *T. gondii* libérés dans le milieu de culture cellulaire, nous avons trouvé que la sortie de *T. gondii* des cellules FPH infectées pouvait être induite par 10 ng/ml de TNF-α, de manière dépendante du temps. Le prétraitement des cellules FPH infectées par BAPTA-AM pour chélater le calcium intra-parasitaire inhibe beaucoup la sortie induite par le TNF-α. Des résultats similaires ont été obtenus en utilisant la cytochalasine D pour bloquer la motilité du parasite avant le test de sortie induite par le TNF-α. De plus, le blocage de l'apoptose de l'hôte par Z-VAD-FMK diminue la sortie induite par le TNF-α, tandis que le blocage de la nécroptose par la nécrostatine-1 a peu d'impact sur la sortie induite par le TNF-α. Les tachyzoïtes évacués avaient un taux de croissance normal et n'ont perdu aucune virulence. Nos résultats suggèrent que les cytokines de l'hôte pourraient influencer le processus lytique cellulaire de *T. gondii* et fournissent de nouvelles informations sur la relation entre TNF-α de l'hôte et pathogenèse de *T. gondii*.

## Introduction

*Toxoplasma gondii* is an obligate intra-cellular parasite that can infect almost all kinds of nucleated cells in warm-blooded animals, leading to toxoplasmosis [6]. Although *T. gondii* commonly causes a self-limiting, usually asymptomatic infection in immunocompetent hosts, it can cause serious disease in the central nervous system in individuals with immunocompromized immune systems, such as AIDS patients, or immature immune systems, such as in newborns [12].

To complete its cellular lytic life cycle, *T. gondii* goes through three distinct processes: active invasion, intra-cellular replication and egress. Host tissue or organ damage is mainly caused at the egress stage when parasites exit from infected cells. The egressed motile parasites further invade neighboring cells to expand their distribution in hosts. Like invasion, parasitic motility plays a critical role in *T. gondii* egress [3]. Moreover, *Toxoplasma* perforin-like protein 1 (*Tg*PLP1) displays structural features necessary for pore formation, which facilitates the natural egress of tachyzoites from infected cells [10]. Other parasitic proteins, such as *Toxoplasma* calcium-dependent protein kinase 1 (*Tg*CDPK1) and *Tg*CDPK3 [11,13], have been reported as molecular regulators for egress of *T. gondii*, both of which function in a calcium (Ca^2+^)-dependent manner. To investigate the molecular mechanisms involved in parasitic egress, researchers have used various chemicals to induce early egress of *T. gondii*. Dithiothreitol (DTT) can activate parasitic nucleoside triphosphate hydrolase (NTPase), leading to depletion of host cell ATP levels and release of tachyzoites from infected cells [19], while another report showed that Ca^2+^ flux induced by DTT may be plausibly linked to NTPase activation and parasitic egress [21]. In a *Staphylococcus aureus* α-toxin-induced egress assay and a nigericin-induced egress assay [5,16], it was found that *T. gondii* also detect potassium (K^+^) efflux from host cells before moving out of infected cells.

Host immune responses induced by *T. gondii* infection are crucial for the establishment of a balanced host-parasite relationship. The typical host immune responses against *T. gondii* infection are production of IL-12, which further activates NK cells to produce IFN-γ, driving the proliferation of type I CD4^+^ and CD8^+^ T cells to produce more IFN-γ. IFN-γ further activates *T. gondii*-infected macrophages to produce nitric oxide (NO) or tumor necrosis factor (TNF), which control replication of the intra-cellular parasite. A number of studies have since provided evidence indicating that certain immune factors, such as IFN-γ [17,18] and NO [9,23], may trigger early egress of *T. gondii* from infected cells. T lymphocytes acting on *T. gondii* infected target cells via death ligand- or perforin/granzyme-dependent cytotoxicity also trigger the egress of infectious parasites with the ability to infect surrounding cells, including the effector cells [18]. Recently, another egress mechanism termed externally triggered egress (ETE) was reported. In this process, external inflammatory factors triggering cells, principally macrophages, elicit egress of *T. gondii* from infected target macrophages. This suggests that other immune factors could serve as a bridge linking host immune responses and parasitic egress [22].

TNF-α is considered a pro-inflammatory cytokine involved in innate immune response. IL-10 knock-out mice infected with *T. gondii* succumbed within the first 2 week of the infection, with high transcription levels of TNF-α [8]. CD154 increased TNF-α production by *T. gondii*-infected macrophages, and neutralization of TNF-α inhibited the effect of CD154 on macrophage anti-*T. gondii* activity. A high TNF-α level contributes to the inflammatory response and to damage of the choroid and retina in ocular toxoplasmosis patients [14]. However, little information is available about the relationship between TNF-α and parasite egress. We hypothesize that in the early stage of *T. gondii* infection, TNF-α may induce egress of tachyzoites from infected cells, which helps parasites invade other cells for mass replication. However, in the late stage of *T. gondii* infection, activated macrophages can eliminate TNF-α-induced egressed *T. gondii*. In this study, we investigated the effects of TNF-α on egress of *T. gondii* from infected human foreskin fibroblast cells (HFFs). TNF-α could trigger early egress of tachyzoites in a time-dependent manner. Intra-parasitic calcium, parasitic motility and the host apoptosis pathway were crucial for TNF-α-induced egress. There were no significant changes in growth rate and virulence of egressed parasites.

## Materials and methods

### Parasites and Reagents

*Toxoplasma* tachyzoites (RH strain, kindly provided by Xun Suo's group at China Agricultural University) expressing yellow fluorescent protein (YFP) in parasitic cytosol were propagated by serial passage in monolayers of human fibroblasts (HFFs). Briefly, HFFs (China Infrastructure of Cell Line Resources, Beijing) were grown in Dulbecco's modified Eagle's medium (HyClone, USA) supplemented with 10% fetal bovine serum (FBS) (HyClone, USA). Parasites were harvested after 3 to 5 days in culture, when the fibroblast host cell monolayer reached 80 to 90% lysis. Recombinant human tumor necrosis factor-alpha (TNF-α) was purchased from Sangon Biotech (Shanghai, China). Other reagents used in this study were Cytochalasin D (Cyto-D), calcium chelator BAPTA-AM, necroptosis inhibitor Necrostatin-1 (Nec-1), and apoptosis inhibitor Z-VAD-FMK (Sigma-Aldrich, USA).

### TNF-α treatment and egress assay

HFFs cultured in 6-well tissue culture plates were used as host cells in this study. Near confluent HFF monolayers were infected with tachyzoites at a multiplicity of infection (MOI) of 0.5 to 1 in a 37 °C incubator for 30 minutes (min) in invasion medium, followed by removal of extracellular parasites through washing with pre-warmed phosphate-buffered saline (PBS). 24 to 36 hours post-infection (hpi), infected cells were treated with 10 ng/mL TNF-α for 3 hours (h) or 6 h. To determine the relationship between intra-cellular development of *T. gondii* and TNF-α-induced egress, HFFs infected with tachyzoites at various times (2 h, 12 h and 24 h) were treated with 10 ng/mL TNF-α for 6 h. The egressed tachyzoites were collected by centrifuging at 1000 rpm for 10 min. Then, tachyzoites were re-suspended with 1 mL cell culture medium and counted using flow cytometry.

In some experimental sets, infected HFFs (about 36 hpi) were pre-treated for 10 min with 1 µM or 5 µM cytochalasin D (Calbiochem, Merck, USA) to block parasitic motility. Intra-parasitic calcium was chelated by BAPTA-AM (20 µM and 50 µM, 20 min) before TNF-α treatment.

To determine whether TNF-α-induced tachyzoite egress is dependent on the host apoptosis or necroptosis pathway, HFFs infected with *T. gondii* for 24∼36 h were pre-treated with 20 µM necrostatin-1 (necroptosis inhibitor) or 10 µM Z-VAD-FMK (apoptosis inhibitor) for 20 min, followed by TNF-α treatment (10 ng/mL, 6 h).

### Virulence detection

RH-YFP tachyzoites were allowed to grow in HFF cells for 36 h in 25 cm^2^ tissue culture flasks and then treated with 10 ng/mL TNF-α for 6 h; egressed parasites were collected at 1000 rpm for 10 min. For the *in vitro* growth assay, egressed tachyzoites were added to HFF cells at an MOI of 0.5-1.0, the number of PV containing different numbers of parasites was determined by microscopic examination 24 hpi. The numbers of rosettes (parasitophorous vacuole, PV) were also counted. Ten fields were directly (without fixing) counted using an Olympus IX 51 fluorescent microscope. Meanwhile, egressed tachyzoites were used to infect C57BL/6 mice intra-peritoneally (10^3^ parasites/mouse, ten mice in each group), and the survival rates after challenge were confirmed. Natural egressed parasites (about 72 hpi) were used in the control group. All animals received humane care, and all testing methods were performed in accordance with the Institutional Guidelines of Anhui Medical University for the Care and Use of Laboratory Animals; the study methods were carried out under the protocol approved by the Institutional Animal Care and Use Committee of Anhui Medical University.

### Statistical analysis

All statistical analyses were processed by SPSS 20.0 Data Editor software (SPSS Inc., Chicago, IL, USA), and all data expressed as means ± SD values. Data analysis methods were *t*-test or one-way analysis of variance (ANOVA). Differences were considered statistically significant at a *p-*value < 0.05.

## Results

### TNF-α induced rapid egress of *T. gondii* tachyzoites from infected HFF cells

**Figure 1:**
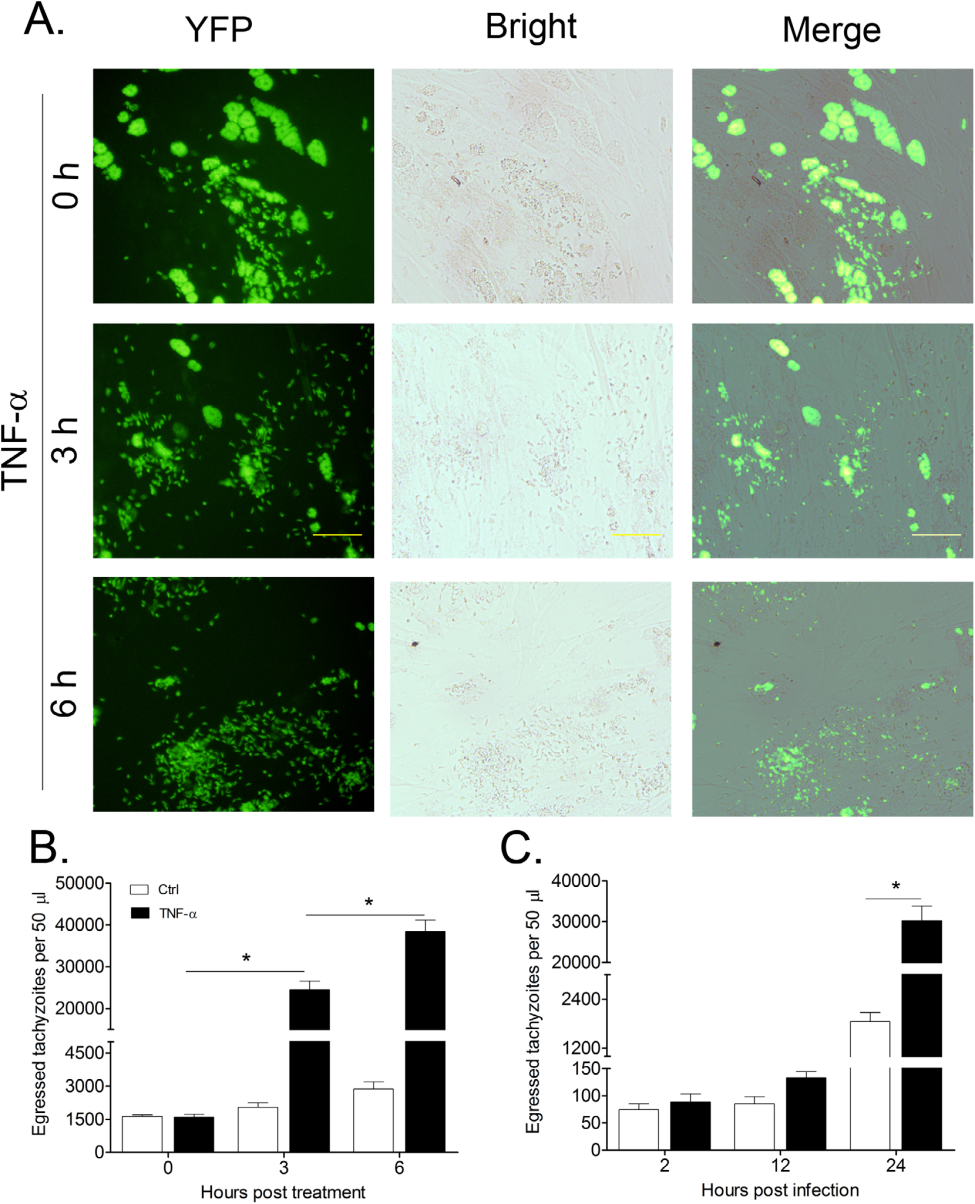
TNF-α triggered egress of tachyzoites from infected HFFs. A and B, HFFs were infected with *T. gondii* (RH strain) for about 36 h, followed by treatment with 10 ng/mL TNF-α for 3 h or 6 h. Egressed parasites were calculated using FACS. C, HFFs were infected with *T. gondii* (RH strain) for different time periods followed by TNF-α treatment (10 ng/mL, 6 h) and an egress assay. Bars represent means ± SDs of five replicates. This experiment was repeated three times with similar results, *p < 0.05. Bar = 50 µm.

To test whether exogenous TNF-α could trigger early egress of *T. gondii*, HFFs infected with tachyzoites for 36 h were treated with 10 ng/mL TNF-α for various time periods. As shown in Fig. 1A and Fig. 1B, treatment of TNF-α could significantly increase the number of tachyzoites in cell culture medium in a time-dependent manner. The amount of free tachyzoites in cell medium in the TNF-α 6 h group was about 20 times higher than the amount of free tachyzoites in the TNF-α 0 h group (38463.6 ± 6044.68 *vs* 1608.2 ± 274.68, *p* < 0.05). Next, we determined whether TNF-α-induced parasite egress was dependent on the intra-cellular development of *T. gondii*. We infected HFFs with tachyzoites for various time periods, and then treated infected cells with TNF-α. As we can see in Fig. 1C, far larger quantities of tachyzoites egressed into medium in groups of 24 hpi (Ctrl *vs* TNF-α, 1850.6 ± 508.15 vs 30273.6 ± 7957.53, *p* < 0.05), indicating that TNF-α-induced egress may depend on the developmental stages of *T. gondii* in host cells.

### TNF-α-induced egress of *T. gondii* depended on intra-parasitic calcium and parasitic motility

**Figure 2:**
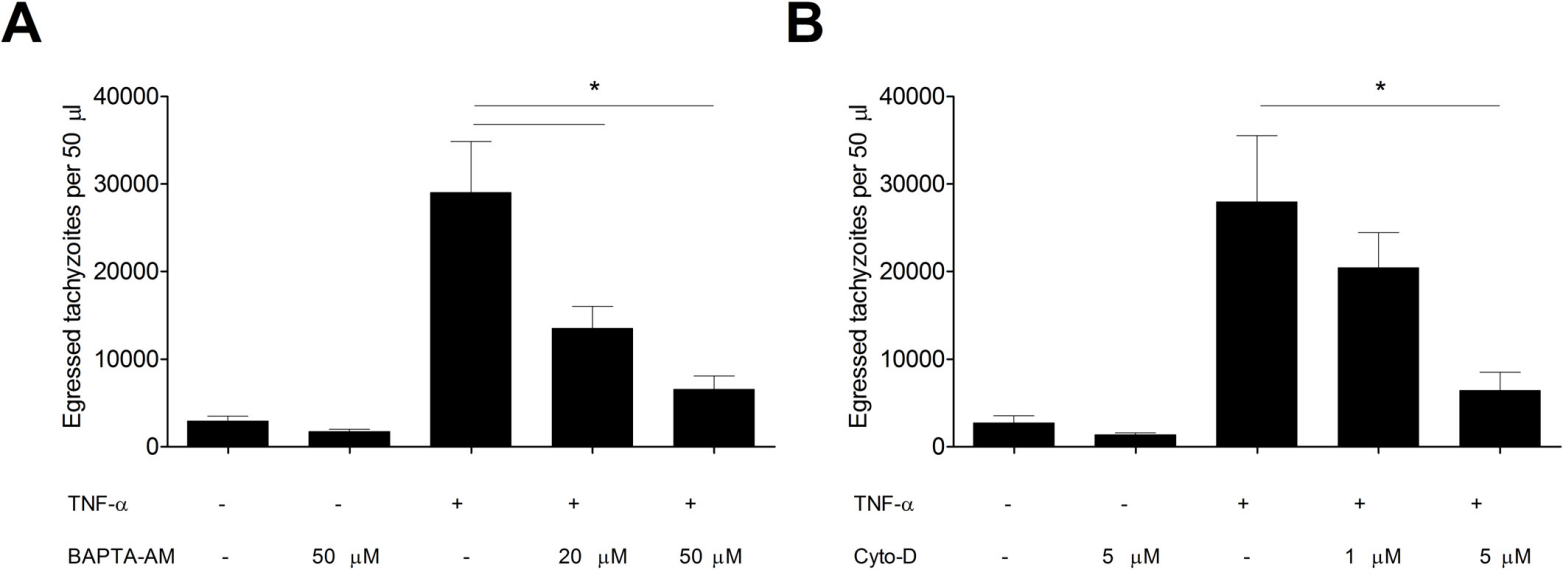
Parasitic motility and intra-parasitic calcium were required for TNF-α-induced egress of *T. gondii*. Infected HFFs (36 h) were pre-treated with BAPTA-AM (A) or Cyto-D (B) before the TNF-α-induced egress assay (10 ng/mL, 6 h, as described in Fig 1). Bars represent means ± SDs of five replicates. This experiment was repeated three times with similar results, *p < 0.05.

As previous studies showed that intra-parasitic calcium plays an important role in egress of *T. gondii*, we performed experiments to determine the essentiality of calcium in TNF-α-induced egress of *T. gondii*. As shown in Fig. 2A, compared to the TNF-α group, pre-treatment with calcium chelator robustly decreased the number of tachyzoites released to cell medium (TNF-α group vs TNF-α/50 µM group, 29038.6 ± 5827.91 *vs* 6568.2 ± 1507.51, *p* < 0.05). Pre-treatment with Cyto-D to block parasitic motility yielded similar results (Fig. 2B), although only a high dose of Cyto-D greatly inhibited TNF-α-induced egress (TNF-α group vs TNF-α/5 µM group, 27942 ± 7560.31 vs 6406.2 ± 2125.72, *p* < 0.05). Thus, TNF-α-induced egress of *T. gondii* relied not only on intra-parasitic calcium but also on parasitic motility.

### Blockade of the apoptosis pathway inhibited TNF-α-induced egress of *T. gondii*

**Figure 3:**
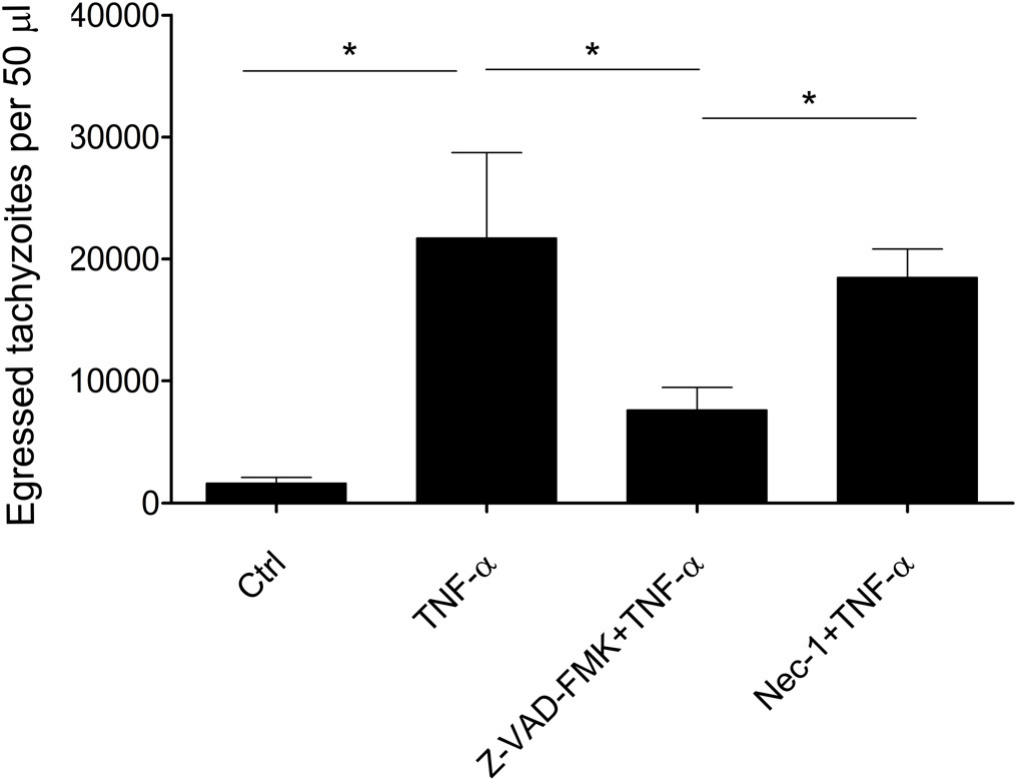
The host cell apoptosis pathway was involved in TNF-α-induced egress of *T. gondii*. Infected HFFs (36 h) were pre-treated with apoptosis inhibitor Z-VAD-FMK (10 µM, 20 min) or necroptosis inhibitor Nec-1 (20 µM, 20 min) before the TNF-α-induced egress assay. Bars represent means ± SDs of five replicates. This experiment was repeated three times with similar results, *p < 0.05.

Previous studies reported that death receptor ligation or exposure to perforin could trigger early egress of *T. gondii* through activation of the host apoptosis pathway [18]. To study the role of the apoptosis pathway in TNF-α-induced egress of *T. gondii*, we pre-treated infected HFFs with apoptosis inhibitor Z-VAD-FMK and found that, compared with the control group (Fig. 3), the egress rate in the Z-VAD-FMK-treated group was significantly lower. Likewise, another cellular death pathway called necroptosis, which is mediated by caspases, has been reported. When infected cells were pre-treated with necroptosis inhibitor necrostatin-1, there was no significant difference between the egress rates of the control group and the treatment group, although the rate in the treatment group was slightly lower.

### Egressed tachyzoites exhibited normal growth and virulence

To detect whether the tachyzoites were infectious after TNF-α-induced egress, we collected egressed parasites to infect HFF cells or inoculate C57BL/6 mice intra-peritoneally. As shown in Table 1, egressed parasites and control parasites developed in HFFs in a similar pattern, indicating no difference in their ability to replicate *in vitro*. The numbers of rosettes formed by egressed parasites and control parasites were of no statistical difference (44.70 ± 5.72 *vs* 46.00 ± 5.56, *p *> 0.05). Meanwhile, there was no significant difference of virulence between egressed parasites and control parasites.

**Table 1:** Egressed parasites displayed normal growth, infectivity and virulence. HFFs were infected with egressed tachyzoites (TNF-α-induced egress) or naturally egressed *T. gondii*. Twenty-four hours later, ten fields were randomly selected and the number of PV containing different tachyzoites was counted using an Olympus IX51 microscope. This experiment was repeated three times with similar results. C57BL/6 mice were infected intra-peritoneally with 10^3^ tachyzoites and the survival rate was evaluated every day. Bars represent means ± SDs of five replicates. This experiment was repeated three times with similar results.

	No. of PV containing different tachyzoites (Tz)				No. of rosettes	Median survival time
			
	2 Tz	4 Tz	8 Tz	16 Tz		
Control	14.60 ± 3.97	20.80 ± 5.40	35.80 ± 8.87	3.40 ± 0.55	46.00 ± 5.56	5.5 days
Egressed	12.80 ± 1.92	17.20 ± 3.03	40.20 ± 9.42	3.60 ± 1.14	44.70 ± 5.72	5.5 days

## Discussion

*T. gondii* can infect all nucleated cells in a diverse array of species, causing tissue or organ damage by lysis of infected host cells through egress. Currently, many researchers have shifted their focus to study the molecular mechanism of how *T. gondii* egress from their host cells [1,4,20]. Previous studies showed that various chemicals, such as DTT, calcium ionophore A23187 and potassium ionophore nigericin, can trigger early egress of *T. gondii* from infected cells [2,5,20]. However, these findings cannot reflect *in vivo* egress of *T. gondii*.

Immunologically, *T. gondii* infection elicits host immune responses, mainly CD8 T cell-mediated immune responses, accompanied by the production of cytokines, such as IL-12 and IFN-γ. It has been reported that IFN-γ could induce early egress of tachyzoites from astrocytes and HFFs [15,17]. In the present study, we found that another inflammatory cytokine TNF-α could also trigger egress of *T. gondii* from HFFs. However, unlike the results of studies showing that IFN-γ-induced egress of tachyzoites from astrocytes and calcium ionophore-induced parasite egress from LLC-MK2 cells did not depend on intra-cellular development of *T. gondii* [2,15], TNF-α-induced egress mainly occurred at 24 hpi. The possible explanation for this phenomenon was that cells used in these three studies were different from each other.

Consistent with previous reports demonstrating that death receptor ligation or exposure to perforin triggering rapid egress of *T. gondii* was mediated through the release of intra-cellular calcium as a consequence of caspase activation early in the apoptotic cascade [18], we found that TNF-α-induced egress was dependent on the availability of intra-parasitic calcium and the apoptosis pathway of host cells. Additionally, we studied the relationship between TNF-α-induced egress and necroptosis. However, necroptosis may not be crucial for the process of TNF-α-induced egress of *T. gondii*, which was partly similar to a previous report showing that IFN-γ-stimulated cell death, which could induce parasitic egress without replication, was not inhibited by necrostatin-1 [17]. As a previous study suggested that *T. gondii* tended to induce apoptosis of infected cells before egress [9], combined with our observation that TNF-α-induced egress mainly occurred at 24∼36 hpi, it may be possible that TNF-α-induced egress mainly relied on the host cell apoptosis pathway. The detailed roles of cellular necroptosis in *T. gondii* egress need further in-depth investigation.

Calcium ionophore induced egress of *T. gondii* shortly after invasion into LLC-MK2 cells (2 hpi), but these egressed parasites could not further multiply inside host cells, indicating that such parasites lost their infectivity [2]. In the present study, no significant changes of growth rate and virulence were detected in TNF-α-induced egressed parasites, which was similar to previous reports demonstrating that reinvasion and normal growth of egressed tachyzoites have been observed in murine peritoneal exudate cells and HFFs [22,23]. It is possible that after host cell invasion, tachyzoites undergo rapidly increasing expression of genes principally devoted to parasite replication, accompanied by decreasing expression levels of genes related to motility and invasion [7]. Although normal growth rate and virulence may benefit parasitic dissemination to other tissues, we cannot exclude the possibility that egressed tachyzoites may be captured by immune-activated macrophages that could eliminate intra-cellular pathogens effectively.

In conclusion, our study found that host cytokine TNF-α could serve as an inducer to trigger egress of *T. gondii* from infected fibroblasts, suggesting a novel role of host inflammatory factors in intra-cellular pathogen infections. Further investigations are needed to confirm whether this inflammatory factor-induced egress of *T. gondii* is found *in vivo*. The effector protein of *T. gondii* interacting with molecules of the host TNF-α signal pathway should be identified, which may provide new directions for anti-toxoplasmosis drug development.

## Conflicts of interest

The authors declare that they have no conflicts of interest in relation to this article.
